# Individualized risk assessment for use of hormone therapy in people with sickle cell disease

**DOI:** 10.1016/j.rpth.2023.102297

**Published:** 2023-12-15

**Authors:** Maureen K. Baldwin, Bethany Samuelson Bannow, Rachel P. Rosovsky, Nancy Sokkary, Lakshmi V. Srivaths

**Affiliations:** 1 Women and Girls with Blood Disorders Learning Action Network, Montclair, New Jersey, USA; 2 Oregon Health & Science University, Portland, Oregon, USA; 3 Division of Hematology, Department of Medicine, Massachusetts General Hospital and Harvard Medical School, Boston, Massachusetts, USA; 4 Department of Obstetrics and Gynecology, Children’s Healthcare of Atlanta/Emory School of Medicine, Atlanta, Georgia, USA; 5 Department of Pediatrics, Gulf States Hemophilia and Thrombophilia Center, McGovern Medical School at the University of Texas Health Science Center, Houston, Texas, USA

We thank Pecker et al. [1] for underscoring the elevated lifetime risk of thrombosis in sickle cell disease (SCD) and the importance of a shared decision-making approach to reproductive health discussions. We missed the opportunity to highlight emerging data regarding thrombosis prevalence and risk factors in this population. In their recently published assessment of prevalence and risk factors for pulmonary embolism (PE) in a pediatric population with SCD using a nationwide administrative database (Pediatric Health Information System), Bala et al. [2] recognized disease state to be a significant factor associated with PE. They found prior use of any hormonal therapy to be more prevalent among pediatric patients with SCD and PE: 8 of 120 (6.7%) with PE vs 494 of 22,511 (2.2%) without PE (odds ratio, 3.91; 95% CI, 2.68, 5.71); however, hormonal therapy was no longer associated with PE after adjusting for multiple factors, including older age, prior acute chest syndrome, central nervous system vasculopathy, prior apheresis, prior use of hydroxyurea, hospital admissions, number of intensive care unit admissions, prior central venous line placement, and obesity/overweight (odds ratio, 1.45; 95% CI, 0.82, 2.58). Another recent large study using an adult patient database (National Inpatient Sample) compared predictors and outcomes after PE between matched patients with and without SCD but did not consider hormone therapy [3]. This gap in knowledge of thrombosis risk of hormonal therapies in SCD could be narrowed by assessing for concurrent hormonal therapy in database studies, based on type if possible, and highlights an urgent need for resources to execute thoughtful multidisciplinary scholarship. As with many other medical conditions (eg, hematologic malignancy, metabolic syndrome, and COVID-19 infection), disease status, age, and comorbidities have varying impacts on individual risk for thrombosis. We agree that there are few situations where a risk-benefit discussion should result in the use of contraceptive doses of estrogen in those with SCD. Progestins are preferred for contraceptive and menstrual indications. These clinical discussions can be facilitated by a hematologist/SCD expert in consultation with a complex family planning specialist, incorporating individualized risk assessment and patient preferences to optimize goals for hormone therapy.
